# *GIGYF1*-disturbed IGF-1R recycling: a potential contributor to autism spectrum disorder pathogenesis?

**DOI:** 10.1172/JCI163553

**Published:** 2022-10-03

**Authors:** Mengen Xing, Qing Zhang, Weihong Song

**Affiliations:** 1 Institute of Aging, Key Laboratory of Alzheimer’s Disease of Zhejiang Province, School of Mental Health and Affiliated Kangning Hospital, The Second Affiliated Hospital and Yuying Children’s Hospital, Wenzhou Medical University;; 2 Oujiang Laboratory, Zhejiang Lab for Regenerative Medicine, Vision and Brain Health, Wenzhou, Zhejiang, China.; 3 Townsend Family Laboratories, Department of Psychiatry, The University of British Columbia, Vancouver, British Columbia, Canada.

## Abstract

Autism spectrum disorder (ASD) is a highly variable and heritable neurodevelopmental disease (NDD) with strong genetic underpinnings. In this issue of the *JCI*, Chen et al. analyzed 2 previously reported, large-scale sequenced ASD cohorts and reported that *GIGYF1* is the second most mutated among ASD risk genes. In this issue of the *JCI*, Chen et al. used a conditional mouse model combined with molecular technologies based on human genetic analyses to determine the critical role of *GIGYF1* in ASD. GIGYF1-deficiency affected the recycling of IGF-1R, thereby suppressing the IGF-1R/ERK signaling pathway. Disruption of GIGYF1 in the developing mouse brain led to social deficits and cognitive impairments. These findings extend our understanding of ASD pathogenesis and provide an avenue for developing potentially effective preventions and treatments for patients with ASD.

## Genetic risks for ASD

Autism spectrum disorder (ASD) is 1 of the most prevalent neurodevelopmental disorders (NDDs) and is characterized by substantial clinical deficits in social and verbal communication, as well as by notably repetitive behaviors (1). ASD is considered highly heritable; around 50%–60% of ASD etiologies are estimated to be genetic (2). While the genetic component of ASD is involved in the development of the disorder, environmental factors such as exposure to physical and chemical factors during pregnancy, maternal infection and immunity, or birth complications, contribute to ASD risk (3, 4). However, our understanding of both genetic and environmental risk factors is still largely limited, and the genetic effects underlying ASD pathogenesis remain unclear.

Pathogenic variants in many high-penetrance genes are extremely rare (2, 5); indeed, the accurate mutation modes, inheritance patterns, and precise phenotypic associations of most ASD risk genes need to be further investigated. In recent years, whole exome sequencing (WES) has been considered one of the most powerful techniques for unveiling the genetic architecture of NDDs (6, 7) like ASD. Particularly, de novo mutations have been implicated as a critical genetic cause and have offered insights into understanding pathogenic genes and cellular mechanisms involved in ASD (8). However, rare, inherited variants in the high-risk genes, based on enrichments of de novo variants, have not been well investigated.

Several ASD-cohort studies are great resources for examining the genetic risks associated with ASD. Of note, 2 important large-scale ASD cohorts (9, 10), the Simons Simplex Collection (SSC) and Simons Foundation Powering Autism Research (SPARK), provide comprehensive information on participants for the assessment of genetic risks for ASD. By combining gene information from the 2 cohorts, in this issue of the *JCI*, Chen et al. (11) discovered the second-most mutated high–confidence ASD risk gene and targeted it for further analysis.

## GIGYF1–induced ASD phenotypic behavior

**Figure 1:**
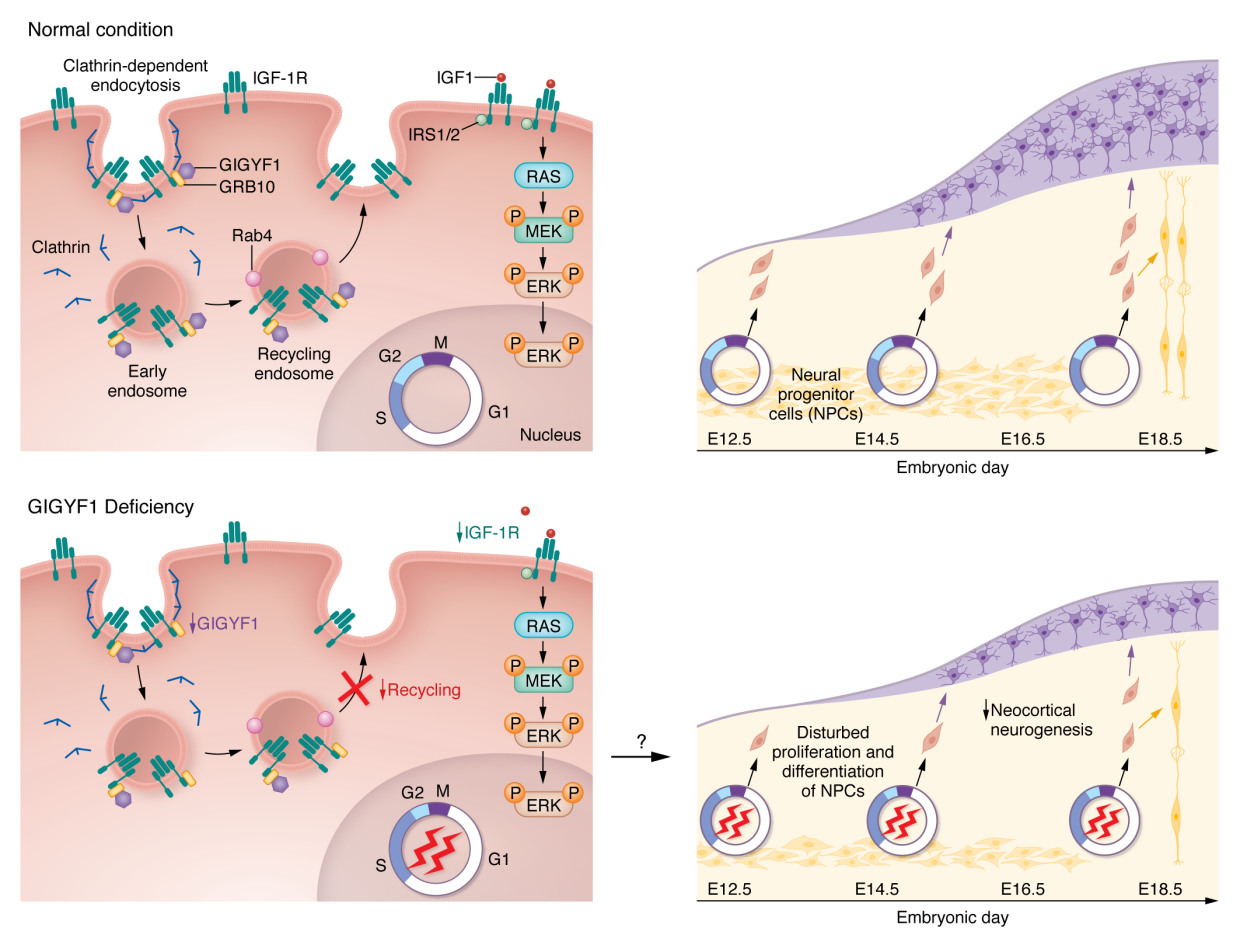
A model for GIGYF1-disrupted IGF-1R signaling as a cause for reduced neocortical neurogenesis. Under normal conditions, GIGYF1 regulates the IGF-1R/ERK signaling pathway, likely through the clathrin-dependent endocytosis and Rab4-mediated recycling of IGF-1R. GIGYF1 forms a complex with GRB10 and IGF-1R and facilitates the internalization of IGF-1R into early endosomes. The complex then transports to Rab4-positive recycling endosomes, and the internalized IGF-1R is recycled to the plasma membrane. IGF1 binds to IGF-1R on the plasma membrane and activates the downstream ERK signaling pathway, which regulates the cell cycle. Normal proliferation, migration, and differentiation of neural progenitor cells (NPCs) are essential for the proper architecture and function of the cerebral cortex. In the scenario of *GIGYF1* haploinsufficiency, the GIGYF1-regulated recycling of internalized IGF-1R to the plasma membrane is reduced. Lower IGF-1R levels on the cell surface reduces IGF-1R/ERK signaling and perturbs the cell cycle dynamics. The disrupted IGF-1R/ERK signaling pathway and cell cycle dynamics may account for the disturbance of NPC proliferation and differentiation caused by *GIGYF1* deficiency. Finally, disturbed NPC proliferation and differentiation reduce neocortical neurogenesis, which may underlie ASD pathogenesis.

Chen and colleagues’ findings provided strong evidence that GIGYF1-deficiency could be a potential contributing factor to ASD pathogenesis (11). It was initially determined that GIGYF1 participates in the insulin-like growth factor receptor (IGF-R) signaling pathway (Figure 1) (12). However, the function and specific role of GIGYF1 and its potential contributions to ASD have not been well characterized. In this issue’s report, Chen et al. illustrated the inheritance modes and mutation patterns of *GIGYF1* variants by analyzing 2 previously reported large-scale ASD cohorts, SSC and SPARK. The paper shows that inherited *GIGYF1* heterozygous likely gene-disruptive (LGD) variants are more common than de novo mutations. These findings emphasize the importance of investigating rare, inherited variants associated with ASD, which will enhance prediction capabilities, especially for families determined to be of high risk. By comparing GIGYF1-associated ASD and NDD phenotypes, the authors demonstrated that *GIGYF1* heterozygous LGD variants might also be associated with ASD or NDD endophenotypes in children without ASD diagnosis. The findings are undoubtedly inspiring for families with ASD with individuals who have the LGD *GIGYF1* variant. However, Chen et al. discuss a concern that the sample size was not large enough to confirm this association. Thus, further studies are needed to validate the present study (11).

A defining feature of autism relates to weakened social and cognitive skills (13). Chen et al. went on to elucidate the mechanism underlying the effect of GIGYF1 on behaviors associated with autism. They introduced a gene-editing technique to conditionally knockout *Gigyf1* (*Gigyf1* cKO) in a mouse model to assess the crucial role of GIGYF1 in ASD. Haploinsufficiency of *Gigyf1* (*Gigyf1*-heterozygous) impaired the social interactions of the mice without causing cognitive impairments, which is consistent with studies involving people with ASD (11). In contrast with *Gigyf1*-heterozygous mice, the complete loss of *Gigyf1* expression in *Gigyf1*-cKO sharply reduced both the social and cognitive skills of the mice. These data indicate that GIGYF1 serves as a critical ASD-risk gene underlying core ASD–related clinical behaviors. However, the phenotype of repetitive behaviors in the *Gigyf1* deficient mice is less certain, which could be an issue of this otherwise reliable mouse model for ASD study (11).

## GIGYF1-disturbed IGF-1R/ERK signaling pathway

A key issue in addressing the function of GIGYF1–disturbed ASD pathogenesis is the interaction between GIGYF1 and the IGF-1R signaling pathway. Although GIGYF1 was indicated to be involved in the IGF-1R signaling pathway, the effect of GIGYF1 on the pathway remains unknown. Chen et al. investigated the effect of GIGYF1 on IGF-1R trafficking and recycling. Knockout of GIGYF1 reduced the level of IGF-1R on the plasma membrane, indicating that GIGYF1 regulates IGF-1R trafficking to the plasma membrane or cell surface. Performing double immunofluorescence and coimmunoprecipitation techniques, the authors demonstrated that GIGYF1 interacts with IGF-1R and GRB10, which is consistent with previous reports of the GIGYF1-GRB10-IGF-1R complex (11, 12). The experimental data also suggest that GIGYF1 regulates IGF-1R trafficking from clathrin-mediated endocytosis to Rab4-mediated recycling to the cell surface. Furthermore, GIGYF1 deletion in different cell lines decreased the phosphorylation of downstream ERK, indicating that GIGYF1 deficiency disturbs the IGF-1R/ERK signaling pathway. Consistently, disturbance of the IGF-1R/ERK signaling pathway was also observed in vivo. The phosphorylation of IGF-1R and ERK was decreased in the cortical lysates from GIGYF1-deficient mice. Notably, the compromised ERK pathway in GIGYF1 knockout cells can be rescued by expressing WT GIGYF1, but not the 3 mutant GIGYF1 variants that were identified in individuals with ASD. These data suggest that GIGYF1 deficiency inhibits IGF-1R/ERK signaling (11).

In the mice with *Gigyf1* deficiency, Chen and colleagues found perturbed p27 and cyclin D1, two critical regulators of the cell cycle (11, 14). They explained that disturbance of the *Gigyf1*-induced IGF-1R/ERK pathway may alter the cell cycle of neural progenitor cells (NPCs) (Figure 1). Indeed, a prolonged S phase was observed in NPCs of both heterozygous and homozygous *Gigyf1*-cKO embryos, which decreased NPC proliferation (11) (Figure 1). Abnormalities in proliferation, migration or differentiation of NPCs could affect the architecture and function of the cerebral cortex in ASD pathogenesis (15, 16). The authors also demonstrated that *Gigyf1* deficiency influenced NPC differentiation, causing reduced distribution of upper cortical neurons in the brains of the mice. Hence, the imbalance of NPC differentiation and proliferation resulting from *Gigyf1* deficiency, in turn, caused disrupted neocortical neurogenesis that is commonly implicated in ASD (11).

However, the regulatory effect of GIGYF1 on IGF-1R recycling was not shown in vivo, and Chen and colleagues’ findings did not provide a clear role of the IGF-1R/ERK signaling pathway in the neocortical neurogenesis that occurs in the development of ASD (11). Thus, future studies are warranted to analyze the interaction between GIGYF1 and IGF-1R in the *Gigyf1*-deficient mice to better understand the regulatory effect of GIGYF1 on IGF-1R recycling. This will help us determine whether GIGYF1 might be an effective candidate for assessing ASD development.

## Concluding remarks and perspectives

Chen et al. mined data from recently sequenced ASD cohorts and identified *GIGYF1* as a high–confidence risk gene for ASD. With subsequent molecular and cellular studies, a genetic animal model, and behavioral tests, they determined the role of GIGYF1 in the risk of ASD pathogenesis. The findings powerfully support the exciting discovery of the GIGYF1-mediated IGF-1R/ERK pathway in ASD development (11).

In conclusion, Chen and colleagues added a critical insight into the complex genetic architecture and biological mechanism of ASD etiology. Their findings also offer possibilities for assessment or prevention of ASD development associated with GIGYF1 deficiency (11). Future analysis of other ASD high-risk genes will be valuable for understanding genetic factors in ASD. With this knowledge, effective measures of assessment and prevention of ASD development, even targeted therapeutics, could be developed.

## Version 1 10/03/2022

Electronic publication
